# Mercurials in Pelvic Cellulitis and Iritis

**Published:** 1865-04

**Authors:** 


### MERCURIALS IN PELVIC CELLULITIS AND IRITIS.

Prof. Simpson, in his Clinical Lectures on the Diseases of Women, says:

"I must leave the question to yourselves to settle how far you will mercurialize your patients. It is ordinarily laid down, more particularly by English authorities, in regard to the treatment of iritis and of almost every form of acute inflammation, that the administration of mercury should be had recourse to as one of the most essential elements in it; and in the treatment of pelvic cellulitis I used formerly to have recourse to it in almost every case as a general rule of practice; and I often have recourse to it still in combination with opium, as in two-grain doses every two hours of the calomel and opium pill of the Pharmacopœia. But I begin more and more to lose faith in its efficacy, for the disease goes on sometimes unchecked even when the mouth is salivated; and I really do not know that we have any certain proof of its power of producing absorption of inflammatory effusions. Ophthalmologists tell us that they can see these effusions beginning to be absorbed in the eye just as the drug begins to exert its constitutional action; but it is assuredly doubtful whether these phenomena stand in the relation of effect and cause, or whether they are not merely coincidences. I have heard Professor John Thomson repeatedly and strongly state that he had occasion to treat forty cases of syphilitic iritis, and having no faith in the reputed power of mercury in the cure of that disease, he treated them without mercury, and succeeded in effecting a cure in all the cases, excepting two, which occurred in the persons of two medical men who had had the misfortune, in the pursuit of their profession, to get their fingers inoculated with syphilitic poison, and who suffered from iritis along with other secondary affection. These two gentlemen had great faith in the power of mercury, and insisted on having it administered to themselves, and in them alone, out of all the forty cases of iritis, did the disease run an unfavorable course and end in loss of vision."—*Boston Journal.*

